# Snake venom L-amino acid oxidases: an overview on their antitumor effects

**DOI:** 10.1186/1678-9199-20-23

**Published:** 2014-06-02

**Authors:** Tássia R Costa, Sandra M Burin, Danilo L Menaldo, Fabíola A de Castro, Suely V Sampaio

**Affiliations:** 1 Department of Clinical, Toxicological and Bromatological Analysis, School of Pharmaceutical Sciences, University of São Paulo (USP), Ribeirão Preto, São Paulo State, Brazil; 2 Faculdade de Ciências Farmacêuticas de Ribeirão Preto, Universidade de São Paulo, Avenida do Café, s/n, B. Monte Alegre, Ribeirão Preto, SP CEP 14040-903, Brasil

**Keywords:** Snake venoms, L-amino acid oxidases, Antitumor effects, Apoptosis

## Abstract

The L-amino acid oxidases (LAAOs) constitute a major component of snake venoms and have been widely studied due to their widespread presence and various effects, such as apoptosis induction, cytotoxicity, induction and/or inhibition of platelet aggregation, hemorrhage, hemolysis, edema, as well as antimicrobial, antiparasitic and anti-HIV activities. The isolated and characterized snake venom LAAOs have become important research targets due to their potential biotechnological applications in pursuit for new drugs of interest in the scientific and medical fields. The current study discusses the antitumor effects of snake venom LAAOs described in the literature to date, highlighting the mechanisms of apoptosis induction proposed for this class of proteins.

## Introduction

The L-amino acid oxidases (LAAOs, EC 1.4.3.2) are flavoenzymes found in such diverse organisms as bacteria, fungi, algae, fish, snails as well as venoms of snakes from the families Viperidae, Crotalidae and Elapidae [1-6].

**Figure 1:**
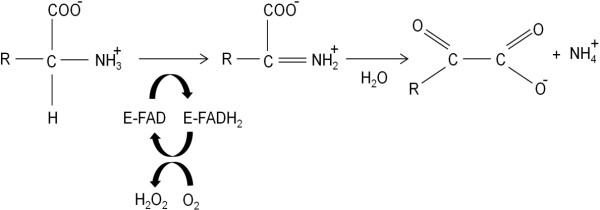
Representation of the reaction catalyzed by L-amino acid oxidases.

Almost all LAAOs described to date are flavoproteins of dimeric structure, with each subunit presenting a non-covalent bond with flavin mononucleotide (FMN) or flavin adenine dinucleotide (FAD). The latter co-factor is commonly found in snake venom L-amino acid oxidases (SV-LAAOs). Flavins present in LAAOs are responsible for the characteristic yellow color of many snake venoms and contribute to their toxicity because of the oxidative stress that results from the production of H_2_O_2_[7]. This feature allows the classification of LAAOs as FAD-dependent oxidoreductases. They are capable of catalyzing the stereospecific oxidative deamination of L-amino acid substrates to α-keto acids. The catalytic cycle, as shown in Figure 1, starts with a reduction half-reaction involving the conversion of FAD to FADH_2_ and the concomitant oxidation of the amino acid into an imino acid, which subsequently undergoes a non-enzymatic hydrolysis releasing α-keto acid and ammonia. Another half-reaction completes the cycle with the oxidation of FADH_2_ by molecular oxygen, producing hydrogen peroxide [8-13].

LAAOs from various sources have been isolated and characterized biochemically, enzymatically and biologically, with the snake venom L-amino acid oxidases (SV-LAAOs) being the most studied enzymes of this family of proteins [2].

In general, SV-LAAOs are homodimers with molecular masses ranging from 120 to 150 kDa in their native form and 50 to 70 kDa in their monomeric forms, and isoelectric point (pI) between 4.4 and 8.12 [2,14]. Interestingly, acidic, neutral and basic forms of SV-LAAOs can coexist in the same snake venom and may present distinct pharmacological properties [15].

Until the 1990s, the studies of SV-LAAOs mainly focused on their physicochemical and enzymatic activities whereas more recent studies have shown that SV-LAAOs present numerous biological and pharmacological effects, such as induction of apoptosis, cytotoxicity, inhibition and induction of platelet aggregation, hemorrhage, hemolysis, edema, as well as microbicidal, antiparasitic and anti-HIV activities [2,7,12,16-21].

Although several SV-LAAOs have been characterized with diverse biological functions, the mechanisms by which these enzymes exert their activities are not fully understood. It is believed that the biological effects of SV-LAAOs is, at least partially, due to the hydrogen peroxide generated during the enzymatic reaction, since the presence of catalase, an agent that degrades H_2_O_2_, can inhibit the action of these enzymes [2].

Nowadays, there is great interest in the clinical use of substances from plants and animals for the treatment of diseases, leading to a search for compounds with modulating actions on the carcinogen metabolism, induction of DNA repair systems and activation or suppression of the cell cycle and apoptosis [22]. Apoptotic processes and cell damage are some of the action mechanisms proposed for many SV-LAAOs, suggesting that these enzymes could be used as models for the development of more effective chemotherapeutic and other antitumor agents [2,13,23,24].

Therefore, this review aims to discuss the cytotoxic effects and the induction of apoptosis in tumor cells by SV-LAAOs. This analysis can serve as an important tool for future research studies on L-amino acid oxidases from snake venoms with antitumor activity.

## Review

### Antitumor potential of SV-LAAOs

Numerous studies of snake venoms show that SV-LAAOs are capable of promoting cytotoxicity in different cell lines, such as S180 (murine sarcoma 180 tumor), SKBR-3 (breast adenocarcinoma), Jurkat (human acute T cell leukemia), EAT (Ehrlich ascites tumor), B16F10 (murine melanoma), PC12 (rat adrenal gland pheochromocytoma), as well as in non-tumor cells (lymphocytes and macrophages) [7]. It is noteworthy that the damage in normal cells is usually negligible when compared to the damage caused in tumor cells [20,25-27]. Although the cytotoxicity mechanisms of SV-LAAOs have not been fully clarified, it is known that lipids present in cell membranes can be damaged by reactive oxygen species (ROS) [28,29]. Considering that membranes of tumor cells present higher concentrations of lipids than normal cells, it is speculated that the hydrogen peroxide produced by LAAOs exerts direct action on the membrane of tumor cells, with lower toxicity on normal cells [30].

Araki *et al.*[31] reported for the first time the apoptosis in vascular endothelial cells caused by hemorrhagic venoms. Shortly afterwards, two other groups of researchers showed that LAAOs from hemorrhagic venoms were primarily responsible for the apoptotic effect on these endothelial cells [32,33]. Since then, many studies have described the apoptotic effect of LAAOs in different cell lines, suggesting this enzyme class is directly linked to the cytotoxic action of venoms [11,13,14,27,33,34].

The effects of SV-LAAOs can be studied by analyzing the cell cycle, which is a set of processes through which a cell passes during its division. This process is divided into two phases: interphase and mitosis, with the interphase being subdivided into G0, G1, S and G2 [35,36]. During the cell cycle, certain stops (checkpoints) occur in order to verify the conditions of the genetic material at the time of cell division; these verifications involve multiple cellular repair proteins (CDK, CKI; CHK), which control the inhibition or the progression of the cycle by different pathways [37]. The generated DNA damage in G1, S or G2 must be repaired as it is the last possible defense against damaged DNA, and if not repaired, the cell proceeds to mitosis and shall initiate the production of defective cells (tumor cells) or undergo cell death by apoptosis [35,36].

The term apoptosis has been proposed by Kerr *et al.*[38] in 1992 to describe the pathway of programmed cell death during cell development, which plays an important role in the development and maintenance of higher organisms. This process is triggered by DNA damage caused by physical, chemical and/or biological agents, and can be defined by various morphological and biochemical characteristics, such as the exposure of phosphatidylserine to the outer leaflet of the plasma membrane, nuclear condensation and the cleavage of chromatin in oligonucleosomal fragments [34,39,40].

Once unleashed, the phenomenon of apoptosis activates molecular events that culminate in the activation of caspases, which are responsible for cell dismantling and death. The process of apoptosis can occur by two major pathways: the intrinsic (mitochondrial) and extrinsic (death receptor). The intrinsic pathway can be triggered by the action of different intracellular stress signals, such as irradiation, chemotherapeutic agents, viruses, bacteria and absence of cell growth factors, which converge on the mitochondria to induce the translocation of cytochrome c and SMAC (second mitochondria-derived activator of caspases) from these organelles to the cytosol, resulting in the presence of APAF-1 and activation of caspase-9. The extrinsic pathway is initiated by the binding of death receptors (DR) – such as Fas/CD95, TNFRI, DR3, DR4, DR5 and DR6 – to their respective ligands. The existing DR are cell surface molecules that have a cysteine-rich extracellular domain and an intracellular domain denominated DD (death domain) [41,42].

The binding of Fas associated with DD (FADD) allows the recruitment of pro-caspase 8 to form the DISC (death-inducing signaling complex). Pro-caspase 8 is self-cleaved and transformed into active caspase 8, and then released into the cytoplasm, where it may act directly on the activation of caspase 3 (executioner phase of apoptosis), or act in the cleavage of Bid molecules that will reach the mitochondria, inducing the release of cytochrome c and SMAC. The cleavage of Bid represents the connection between the extrinsic and intrinsic pathways of apoptosis [41,43].

The mitochondrial pathway is regulated by members of the Bcl-2 family, which are cytoplasmic proteins capable of integrating signals of survival or cell death generated in the intra- and extracellular medium [44]. This family is divided into two classes: anti-apoptotic proteins (Bcl-2, Bcl-x_L_, Bcl-w, A1 and Mcl-1), whose function is to protect cells from death, and pro-apoptotic proteins (Bax, Bak, Bad, Bid, Bmf etc.) that sensitize or lead cells to apoptosis [44]. The executioner pathway of apoptosis is common to both initiating pathways and is characterized by the activation of effector caspases, namely caspase-3, −6 and −7, and the cell-dismantling characteristic of apoptosis [45-47]. The balance of the interactions between pro- and anti-apoptotic proteins may define the occurrence of cell death.

Numerous studies have reported that apoptotic processes induced by LAAOs are partially explained by the generation of hydrogen peroxide (H_2_O_2_), a reactive oxygen species (ROS) that accumulates on the surface of cell membranes. It is widely accepted that increasing ROS concentrations promotes mitochondrial derangements that cause cell death [2,7,11,13,23,27,32-34,48,49]. In this context, several studies with SV-LAAOs evaluated their cytotoxic effects in the presence of catalase (known for its ability to degrade H_2_O_2_ to H_2_O and O_2_), revealing that in fact the toxic action of SV-LAAOs is practically annulled by this agent [2,7,50].

To evaluate the cytotoxic activity of SV-LAAOs, most studies make use of the colorimetric method for cytotoxicity proposed by Mosmann [51]. Ahn *et al.*[25] showed that the LAAO isolated from *Ophiophagus hannah* (king cobra) venom is cytotoxic for stomach cancer cells (SNU-1). LAAOs from *Agkistrodon acutus* (ACTX-6) and *Bungarus fasciatus* (BF-LAAO) showed cytotoxic effects on A549 cells (lung adenocarcinoma), with ACTX-6 presenting an IC_50_ of 20 μg/mL [23,49]. Alves *et al.*[27] assessed the cytotoxic effects of an LAAO isolated from *Bothrops atrox* venom (named BatroxLAAO) on various tumor cell lines, such as HL-60 (IC_50_ 50 μg/mL), PC12, B16F10 and JURKAT (IC_50_ of 25 μg/mL for the three cell lines). Also, in the presence of catalase (150 U/mL), BatroxLAAO did not induce significant cell death on any of the tumor cell lines tested [13].

One study revealed the toxin Bl-LAAO from *Bothrops leucurus* venom presented a cytotoxic effect on the tumor cell lines MKN-45 (stomach cancer), RKO (colorectal cancer) and LL-24 (human fibroblasts), whereas around 25% of this cytotoxicity was inhibited in the presence of catalase (100 μg) [19].

Bregge-Silva *et al.*[52] evaluated the cytotoxic effect of an LAAO (denominated LmLAAO) isolated from *Lachesis muta* snake venom on AGS (gastric adenocarcinoma) and MCF-7 (breast tumor) cells, with IC_50_ of 22.7 μg/mL and 1.41 μg/mL, respectively. The catalase (0.1 mg/mL) completely abolished the cytotoxic effects of LmLAAO on MCF-7 tumor cells.

Several SV-LAAOs isolated from different snake venoms have been described as able to induce cell death in different cell lines [14,20,53,54]. A study with the LAAO isolated from *Agkistrodon halys* snake venom demonstrated the apoptotic action of this protein on murine lymphoblastic leukemia cells (L1210) by quantitatively analyzing the DNA fragmentation after treatment of cells with the protein. Twenty-four hours after treatment, death by necrosis was observed, suggesting that higher amounts of H_2_O_2_ were released during the enzymatic reaction. When cells were treated concomitantly with catalase, cell viability was not fully restored, indicating that the apoptotic activity of LAAOs cannot be explained completely by the generation of hydrogen peroxide [32].

Torii *et al.*[33] evaluated the apoptotic effects of Apoxin I, an LAAO from *Crotalus atrox* snake venom. Authors showed that Apoxin I at 10 μg/mL of this venom induced condensation and fragmentation of chromatin in human umbilical endothelial cells, HL-60, A2780 (human ovarian carcinoma) and NK-3 (rat endothelial cells). At a concentration of 2.5 μg/mL, Apoxin I induced oligonucleosomal DNA fragmentation in HL-60; however, at lower concentrations, the toxin did not induce apoptosis in this lineage. This study also showed that the induction of apoptosis was completely abolished when the LAAO was inactivated by changes in temperature (70°C) or in the presence of catalase. It was also found that in the presence of a membrane antioxidant (trolox), the Apoxin I was not able to induce apoptosis in the tested cell lines. These findings suggest that the apoptotic effect caused by Apoxin I is related to the catalytic activity of the enzyme, which is responsible for the production and release of H_2_O_2_ that may be related to the oxidation of the cell membrane [33].

ACL LAO, isolated from *Agkistrodon contortrix laticinctus* venom, was also capable of inducing apoptosis in HL-60 cells. Twenty-four hours after treatment with 25 μg/mL of the toxin, a typical pattern of DNA fragmentation in apoptotic cells was observed [14]. Low concentrations of another protein of this class, the VB-LAAO from *Vipera berus berus* venom, induced apoptosis in K562 and HeLa tumor cell lines, whereas at higher concentrations, this enzyme also induced necrosis in K562 cells [55].

To examine the apoptotic and necrotic effects induced by SV-LAAOs, two flow cytometry methods have been employed: Annexin V FITC and HFS (hypotonic fluorescent solution, containing 50 μg/mL of propidium iodide in 0.1% sodium citrate plus 1.0% Triton X-100). Cells in early apoptosis are positive for annexin V and negative for propidium iodide (PI), which indicates phosphatidylserine externalization and membrane integrity. The assessment of DNA content detected by the HFS method considers the incorporation of PI in isolated nuclei compatible with the diploid content, whereas apoptotic nuclei appear in the hypodiploid region of the histogram due to the fragmentation of the nucleus or the greater condensation of chromatin [56].

The apoptotic and necrotic effects of BatroxLAAO were analyzed by flow cytometry. This toxin induced cell death processes in different tumor cell lines, such as JURKAT, B16F10, PC12 and HL-60. The B16F10 and PC12 cell lines presented death by apoptosis (AV+), while JURKAT cells displayed death by necrosis (27% necrotic cells) [27]. In HL-60, 50 μg/mL BatroxLAAO showed apoptotic effect in 28.6% and necrotic effect in 14.2% of cells, maintaining a cell viability of approximately 57% [13]. These data corroborate the study by Ande *et al.*[34], which evaluated the effects of CR-LAAO from *Calloselasma rhodostoma* venom on the viability of JURKAT leukemia cells and the influence of catalase on apoptosis induction. CR-LAAO induced necrosis (PI+) in JURKAT cells in a dose-dependent manner. However, in the presence of catalase, the number of necrotic cells was drastically reduced, and a corresponding increase in the number of apoptotic cells (AV+) was observed, probably related to the catalase treatment.

Other studies have demonstrated the induction of apoptosis promoted by SV-LAAOs by the increased percentages of hypodiploid nuclei in tumor cell lines. Wei *et al.*[49] showed that after 12 hours of treatment with BF-LAAO, the concentrations of 0.03, 0.1, 0.3, 1.0 and 3.0 μg/mL induced respective apoptosis proportions of 3.7, 6.6, 14.0, 32.4 and 41.2% in A549 cells. Burin *et al.*[20] conducted tests to assess the effect of BpirLAAO (from *Bothrops pirajai* venom) on HL-60 and HL-60.Bcr-Abl tumor cell lines. Their results showed a dose-dependent increase in the percentage of hypodiploid nuclei 18 hours after treatment.

Furthermore, to assess whether SV-LAAOs induced apoptosis by the intrinsic (mitochondrial) or extrinsic (death receptor) pathway, some studies evaluated the detection of caspases 3, 8 and 9. Alves *et al.*[27] reported the activation of caspases 3 and 9 24 hours after treatment of PC12, HL-60, JURKAT and B16F10 cell lines with BatroxLAAO. In relation to BpirLAAO, Burin *et al.*[20] observed activation of caspases 3, 8 and 9 18 hours after treatment of HL-60 and HL-60.Bcr-Abl cell lines with BpirLAAO. These results suggest that SV-LAAOs may act in the activation of the intrinsic and extrinsic pathways of apoptosis.

Currently, molecular biology assays such as the combination of reverse transcription with quantitative real-time polymerase chain reaction (RT-qPCR) have contributed much to the study of the apoptotic potential of SV-LAAOs. The detection of the expression of pro- and anti-apoptotic genes assists in determining the apoptosis pathway (intrinsic or extrinsic) activated by these enzymes. The LAAO from *Agkistrodon acutus* venom (named ACTX-8) induced apoptosis in HeLa cells mediated by the mitochondrial pathway, which was detected by verifying the translocation of Bax and Bad from the cytosol to the mitochondria [57].

Few studies have been conducted to assess the effects of SV-LAAOs on the cell cycle progression. de Melo Alves-Paiva *et al.*[13] evaluated the cycle modulation and the induction of apoptosis in HL-60 cells treated with BatroxLAAO, showing that this toxin induced a delay in the G0/G1 phase. The authors suggested that this delay may prevent the initiation of DNA synthesis and, consequently, the replication of tumor cells, which could represent another possible mechanism by which SV-LAAOs display their antitumor effects. Similar results were observed when LAAO was isolated from *Agkistrodon acutus* venom (ACTX-6), which promoted a 15% increase of A549 cells in the G0/G1 phase compared to the untreated group [23]. K562 and U937 cells presented that same delay profile in G1 and decreased number of cells in the G2/M phase after treatment with drCT-I isolated from *Daboia russelli russelli* venom [58].

## Conclusions

Apoptosis, cell damage and alteration in cell cycle processes may be induced by SV-LAAOs in different tumor cell lines, which emphasizes the antitumor potential of this class of toxins. Some of these SV-LAAOs and the tumor cells in which they were tested are summarized in Table 1.

**Table 1:** Summary of some SV-LAAOs and the tumor cell lines in which they were tested

**Snake species**	**LAAO**	**Tumor cell lines**	**Methodology**	**References**
*Agkistrodon acutus*	ACTX-6	A549	MTT	[23]
	ACTX-8	HeLa	MTT, DNA fragmentation	[57]
			Activation of caspases 3 and 9	
*Agkistrodon contortrix laticinctus*	ACL LAO	HL-60	DNA fragmentation	[14]
*Agkistrodon halys*		L1210	DNA fragmentation	[32]
		MOLT-4		
		HL-60		
*Agkistrodon halys pallas*		A549	DNA fragmentation	[59]
*Bothrops atrox*	BatroxLAAO	HL-60	MTT	[13,27]
		PC12	Annexin V	
		B16F10	Activation of caspases	
		Jurkat		
*Bothrops moojeni*	BmooLAAO-I	HL-60 and EAT	MTT and DNA fragmentation	[60]
*Bothrops pirajai*	BpirLAAO-I	S180	MTT	[20,26]
			DNA fragmentation	
			HFS	
			Activation of caspases 3, 8 and 9	
		SKBR3		
		HL-60		
		HL-60.Bcr-Abl		
		EAT		
*Bungarus fasciatus*	BF-LAAO	A549		[49]
*Calloselasma rhodostoma*	CR-LAAO	Jurkat		[34]
*Crotalus atrox*	Apoxin-I	HL-60	DNA fragmentation	[33,54]
		A2780		
		HUVEC		
		KN-3		
*Eristocophis macmahoni*	LNV-LAO	MM6	DNA fragmentation	[53]
*Ophiophagus hannah*		SNU-1	MTT	[25,61]
		B16F10	DNA fragmentation	
		MCF-7	Activation of caspases	
		A549		
*Vipera berus berus*		HeLa and K562	DNA fragmentation	[55]

The mechanisms by which SV-LAAOs induce apoptosis are still not known, but studies suggest that the H_2_O_2_ produced during the enzymatic reaction, the activation of caspases and/or the interaction of LAAOs with membrane receptors may be involved in this cell death process.

Conducting new studies to elucidate the action mechanisms of SV-LAAOs are necessary to develop novel therapeutic strategies with more directed actions, which would result in more effective chemotherapeutic and antitumor agents.

## Competing interests

The authors declare that there are no competing interests.

## Authors’ contributions

TRC and SMB contributed equally to the conceiving and writing of this review. DLM participated in the writing and FAC and SVS supervised and critically discussed the review. All authors read and approved the final manuscript.
